# The complete mitogenome of *Hemiculter leucisculus* (Basilewsky, 1855) in Hainan Island and its phylogenetic status

**DOI:** 10.1080/23802359.2022.2040390

**Published:** 2022-02-20

**Authors:** Congqiang Luo, Peng Chen, Jibin Zhu, Zemin Ma

**Affiliations:** a Changde Research Center for Agricultural Biomacromolecule, Hunan University of Arts and Science, Changde, China; b Changde Win-win Environmental Consul Ting Service Co. LTD, Changde, China; c Changde Municipal Waterworks Co. LTD, Changde, China

**Keywords:** *Hemiculter leucisculus*, mitogenome, phylogeny

## Abstract

The sharpbelly, *Hemiculter leucisculus* (Basilewsky, 1855) is a small cyprinid fish that has a wide distribution in East Asia. In this study, we characterized the complete mitochondrial genome of *H. leucisculus* in Hainan Island using Illumina MiSeq platform. The mitogenome contained 16,621 bp with AT content of 56.2%. The mitogenome of *H. leucisculus* comprised 13 protein-coding genes, 22 transfer RNA genes, 2 ribosomal RNA genes, and one control region (D-loop). Phylogenetic analyses indicated that *H. leucisculus* in the Hainan Island formed independent lineage and the species of *H. leucisculus* might be a paraphyletic taxon.

## Introduction

The sharpbelly, *Hemiculter leucisculus* (Basilewsky, 1855), is a small cyprinid fish that has a wide distribution in the drainage basins of China, Korea island, Japan, Mongolia, and Russia (www.fishbase.org). In China, *H. leucisculus* widely occupies various freshwater habitats, such as rivers, lakes, reservoirs, and even pools (Luo and Chen 1998). Habitat generalist and wide distribution ranges of the *H. leucisculus* populations make this species possibly show complex phylogenetic status. Chen et al. (2017) demonstrated that *H. leucisculus* populations in southern China showed deep divergence with the Yangtze River populations and could be regarded as a cryptic species. Herein, we reported *H. leucisculus* mitogenome from Hainan Island and attempted to infer its phylogenetic status within genus *Hemiculter*.

Specimen of *H. leucisculus* was collected from the farmers market of Ledong County, Hainan Province, China (18.75219 N, 109.17497E) on 27 October 2019 and was carefully distinguished this species according to the ichthyography by Luo and Chen (1998). We extracted total genomic DNA from fin tissues using a Genomic DNA Isolation Kit (QiaGene, Germany). The sample and the total DNA was stored in the fish collection of Hunan University of Arts and Science (www.huas.edu.cn, Congqiang Luo and blackball@yeah.net) under the voucher numbers HL2019001. We sequenced the complete mitochondrial genome using the Illumina MiSeq platform (Illumina Inc, San Diego, CA, U.S.A.) and assembled the raw sequence reads into contigs using SPAdes 3.9.0. The complete mitochondrial genomes were finally obtained utilizing the contigs in SOAPdenovo (Luo et al. 2012) and were annotated using GeSeq (Tillich et al. 2017) with NCBI reference sequences.

Mitogenome length and AT content for the individual from Hainan Island are 16,621 bp and 56.2%, respectively (GenBank accession: MZ520999). This mitogenome showed similar AT contents with that in previous reported *H. leucisculus* mitogenome sampled from the Heilongjiang Province, China (GenBank accession: KF647872; Dong et al. 2015) and the Zhejiang Province, China (GenBank accession: KF956522; Xiang et al. 2016). The complete mitochondrial genome comprised of 13 protein-coding genes (PCGs), 2 ribosomal RNA (rRNA) genes (12S rRNA and 16S rRNA), 22 transfer RNA genes and a control region (D-loop). The 13 PCGs of the mitogenome had same initiating codon and stop codon with the *H. leucisculus* mitogenome from the Zhejiang Province (Xiang et al. 2016) but showed different stop codon with *H. leucisculus* mitogenome from the Heilongjiang Province in *ND5* gene (Dong et al. 2015).

**Figure 1.**
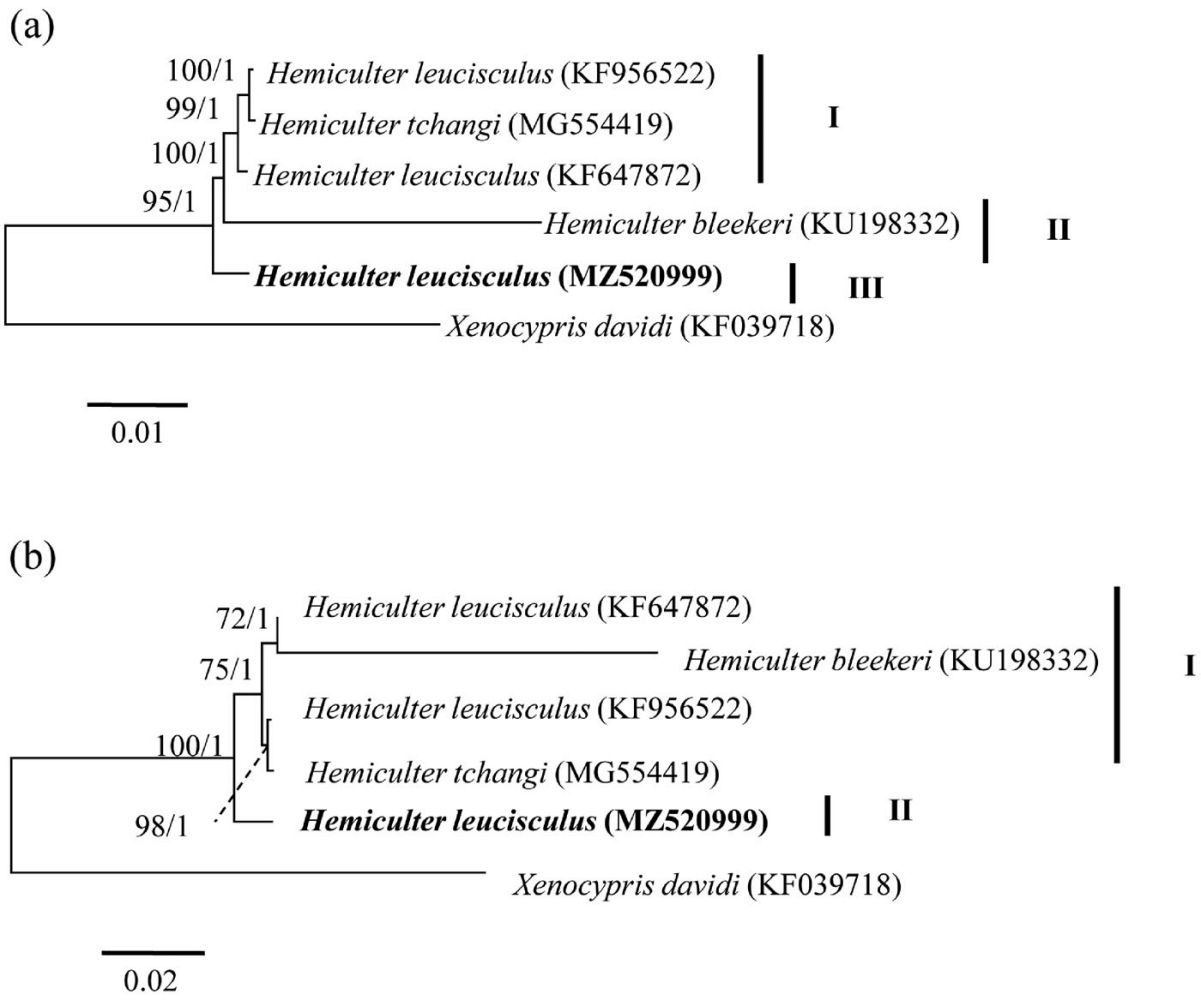
Maximum-likelihood trees showing the phylogenetic relationships among *Hemiculter* species based on 13 protein-coding genes (a) and amino acids (b), respectively. Values on branches indicate bootstrap values from maximum-likelihood analysis and posterior probability from Bayesian inference.

To infer the phylogenetic status of *H. leucisculus* populations in the Hainan Island, we downloaded published *Hemiculter* mitogenomes and built Bayesian inference (BI) and maximum-likelihood (ML) trees using the combined DNA sequences of 13 PCGs and corresponding amino acids in RAXML-VI-HPC (Stamatakis 2006) and MRBAYES 3.2 (Ronquist and Huelsenbeck 2003), respectively. *Xenocypris davidi* (Genbank nos: KF039718) from Xenocyprinae was selected as an outgroup. Optimal nucleotide substitution model of GTR + I + G for phylogenetic analyses were selected using the Akaike information criterion in MRMODELTEST version 2.3 (Nylander 2004). For the analyses with amino acids, we allowed MrBayes to estimate the best fixed-rate amino acid matrix (i.e. prset aamodelpr = mixed) and used WAG model inferred from to construct ML tree. BI and ML trees based on 13 PCGs consistently obtained three lineages (Figure 1(a)), while BI and ML trees on the basis of amino acids congruously yielded two lineages (Figure 1(b)). The discordant result between the two molecular types was the placement of *H. bleekeri* (Figure 1). All trees showed that *H. leucisculus* in the Hainan Island formed an independent lineage and *H. leucisculus* was a paraphyletic taxon (Figure 1). However, further studies including morphology and genetics were needed to demonstrate this point.

## Data Availability

The genome sequence data that support the findings of this study are openly available in GenBank of NCBI at (https://www.ncbi.nlm.nih.gov/) under the accession no. MZ520999. The associated BioProject, SRA, and Bio-Sample numbers of specimen are PRJNA745010, SRR15080965 and SAMN20130455, respectively.

## References

[CIT0002] Chen W, Zhong Z, Dai W, Fan Q, He S. 2017. Phylogeographic structure, cryptic speciation and demographic history of the sharpbelly (*Hemiculter leucisculus*), a freshwater habitat generalist from southern China. BMC Evol Biol. 17(1):216. 28899345 10.1186/s12862-017-1058-0 PMC5596851

[CIT0003] Dong F, Tong G, Kuang Y, Zheng X, Sun X. 2015. The complete mitochondrial genome sequence of *Hemiculter leucisculus*. Mitochondrial DNA. 26(5):747–748. 24460158 10.3109/19401736.2013.848351

[CIT0004] Luo R, Liu B, Xie Y, Li Z, Huang W, Yuan J, He G, Chen Y, Pan Q, Liu Y, et al. 2012. SOAPdenovo2: an empirically improved memory-efficient short-read de novo assembler. Gigascience. 1(1):18. 23587118 10.1186/2047-217X-1-18 PMC3626529

[CIT0005] Luo Y, Chen Y. 1998. Culterinae. Fauna Sinica, Osteichthyes, Cypriniformes II. Beijing: Science Press.

[CIT0006] Nylander J. 2004. MrModeltest v2. Program distributed by the author. Nylander JAA, editor. Uppsala: Evolutionary Biology Centre, Uppsala University.

[CIT0007] Ronquist F, Huelsenbeck JP. 2003. MrBayes 3: Bayesian phylogenetic inference under mixed models. Bioinformatics. 19(12):1572–1574. 12912839 10.1093/bioinformatics/btg180

[CIT0008] Stamatakis A. 2006. RAxML-VI-HPC: maximum likelihood-based phylogenetic analyses with thousands of taxa and mixed models. Bioinformatics. 22(21):2688–2690. 16928733 10.1093/bioinformatics/btl446

[CIT0010] Tillich M, Lehwark P, Pellizzer T, Ulbricht-Jones ES, Fischer A, Bock R, Greiner S. 2017. GeSeq–versatile and accurate annotation of organelle genomes. Nucleic Acids Res. 45:6–11. 10.1093/nar/gkx391 PMC5570176 28486635

[CIT0011] Xiang D, Ai W, Peng X, Qiu J, Chen S. 2016. Complete mitogenome of *Hemiculter leucisculus* (Cyprinidae: Cultrinae). Mitochondrial DNA A DNA Mapp Seq Anal. 27(1):145–146. 24450718 10.3109/19401736.2013.878916

